# An Efficient Single Phase Method for the Extraction of Plasma Lipids

**DOI:** 10.3390/metabo5020389

**Published:** 2015-06-17

**Authors:** Zahir H. Alshehry, Christopher K. Barlow, Jacquelyn M. Weir, Youping Zhou, Malcolm J. McConville, Peter J. Meikle

**Affiliations:** 1 Baker IDI, Heart and Diabetes Institute, Melbourne, VIC 3004, Australia; E-Mails: Zahir.Alshehry@bakeridi.edu.au (Z.H.A.); Christopher.Barlow@bakeridi.edu.au (C.K.B.); jacqui.weir@bakeridi.edu.au (J.M.W.); zhouyouping@yahoo.com (Y.Z.); 2 King Fahad Medical City, Riyadh 11525, Saudi Arabia; 3 The Bio21 Institute of Molecular Science and Biotechnology, University of Melbourne, Melbourne, VIC 3010, Australia; E-Mail: malcolmm@unimelb.edu.au

**Keywords:** lipidomics, 1-butanol/methanol extraction, mass spectrometry

## Abstract

Lipidomic approaches are now widely used to investigate the relationship between lipid metabolism, health and disease. Large-scale lipidomics studies typically aim to quantify hundreds to thousands of lipid molecular species in a large number of samples. Consequently, high throughput methodology that can efficiently extract a wide range of lipids from biological samples is required. Current methods often rely on extraction in chloroform:methanol with or without two phase partitioning or other solvents, which are often incompatible with liquid chromatography electrospray ionization-tandem mass spectrometry (LC ESI-MS/MS). Here, we present a fast, simple extraction method that is suitable for high throughput LC ESI-MS/MS. Plasma (10 μL) was mixed with 100 μL 1-butanol:methanol (1:1 v/v) containing internal standards resulting in efficient extraction of all major lipid classes (including sterols, glycerolipids, glycerophospholipids and sphingolipids). Lipids were quantified using positive-ion mode LC ESI-MS/MS. The method showed high recovery (>90%) and reproducibility (%CV < 20%). It showed a strong correlation of all lipid measures with an established chloroform:methanol extraction method (R^2^ = 0.976). This method uses non-halogenated solvents, requires no drying or reconstitution steps and is suitable for large-scale LC ESI-MS/MS-based lipidomic analyses in research and clinical laboratories.

## 1. Introduction

Dyslipidemia is strongly associated with cardiovascular diseases [1,2,3], insulin resistance and diabetes [4,5]. Commonly used clinical lipid measurements such as low density lipoprotein-cholesterol (LDL-C), high density lipoprotein-cholesterol (HDL-C) or triacylglycerols, provide only limited information as they fail to measure the individual lipid species that make up the lipoprotein pools. Advances in mass spectrometry have allowed the examination of hundreds of lipids, often in a single experiment [6,7]. It has been reported that individual lipids are associated with the development of diseases including insulin resistance and diabetes [8], cardiovascular disease [9], hypertension [10], cancer [11] cystic fibrosis [12], as well as with smoking [13] and drug action [14]. Furthermore, individual lipid species within a class often show differential associations with a disease state [6]. Lipidomic analysis of plasma represents a new approach that can inform on disease processes [15] and potentially identify useful biomarkers to diagnose and assess disease risk [6].

Lipidomic analysis of population-based studies provides the potential to determine the association of individual lipid species with disease progression and outcomes. The concentration of lipid species are closely associated with many traditional risk factors and so determination of independent associations often requires adjustment for a large number of covariates. Additionally the analysis of hundreds of lipids necessitates suitable statistical corrections to account for problems associated with multiple comparisons [16,17,18]. Consequently, such studies require the analysis of hundreds to thousands of samples to ensure sufficient statistical power if independent associations are to be established. Such studies necessitate the lipidomic methodology be simple, robust and high-throughput. Furthermore, these characteristics are critical if plasma lipid profiling is to find clinical application.

There are several methods suitable for the extraction of lipids from plasma. Possibly the most widely used is the “Folch” method [19]. Briefly, this is a two-phase liquid-liquid extraction utilizing 2:1 (v/v) chloroform:methanol in which the majority of lipids partition into the lower organic phase. Several additional two-phase liquid-liquid extraction methods have been reported with several modifications including the Bligh-Dyer [20] and acidic Folch [21]. A key disadvantage of these approaches is that the collection of the lower organic phase is cumbersome resulting in increased processing time, and reduced reproducibility and throughput. Matyash and co-workers have suggested that the use of methyl-tert-butyl ether (MTBE) may overcome this problem as the lipid rich organic layer settles above the aqueous phase [22]. More recently, an automated method utilizing 1-butanol/methanol in a two-phase system (BUME method) has been described [23] for use in direct infusion “shotgun” mass spectrometry analysis. As in the MTBE method, the lipids partition into the upper phase, simplifying their collection. Nevertheless, care must still be taken to avoid the interface between the two phases in both methods. Recently three of these extraction methods (Folch, Bligh Dyer and the MTBE methods) together with the acidified Bligh Dyer and a hexane-isopropanol method were compared for their ability to extract lipids from human low-density lipoprotein [24]. These experiments demonstrated that the recoveries of the major lipid classes were similar although for lower abundant classes there was significant variation between methods with no single method showing optimal performance for all lipid classes [24].

The mass spectrometry approach taken, places specific demands on the extraction methodology employed. Shotgun lipidomics requires a high degree of sample preparation to ensure the removal of unwanted components such as salts and polar metabolites, which may suppress lipid ions. In contrast, lipidomic approaches utilizing reverse phase liquid chromatography electrospray ionization- tandem mass spectrometry (LC ESI-MS/MS), require less stringent sample preparation as polar impurities are eluted at the solvent front and do not interfere with the MS analysis of the lipids. We have previously described a single-phase lipid extraction method [6,7] suitable for use with reverse phase LC ESI-MS/MS analysis using chloroform and methanol. A shortcoming of this method was that following initial extraction it required the removal of the extraction solvent and reconstitution in a mixture of 1-butanol and methanol to give satisfactory chromatographic performance. Here we report on a new single-phase lipid extraction using 1-butanol/methanol (1:1 v/v) that does not require removal of the solvent and reconstitution prior to reverse-phase LC ESI-MS/MS analysis. The method is extremely rapid allowing for high throughput sample preparation, shows a high level of recovery (>90%) for a wide range of polar and non-polar lipids, and is highly reproducible.

## 2. Results

### 2.1. Recovery of Lipids

**Figure 1:**
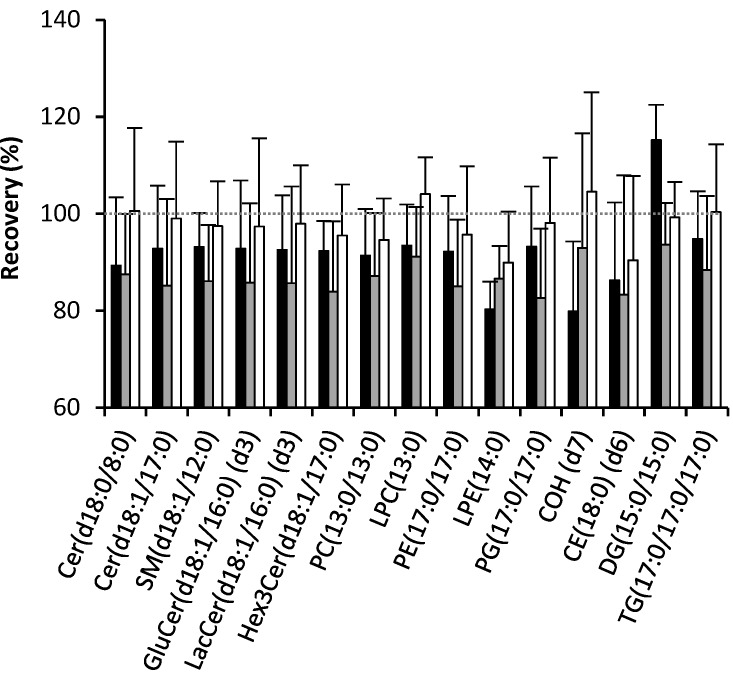
Recovery of lipids with the different extraction methods. The recovery calculated the percent of recovery of the method (*n* = 5) against its spiked equivalent method (*n* = 5). The black bars represent the recovery of chloroform/methanol against spiked chloroform/methanol, the gray bars represent the recovery of 1-butanol/methanol (3:1 v/v) against spiked 1-butanol/methanol (3:1 v/v) and the white bars represent the recovery of 1-butanol/methanol (1:1 v/v) against spiked 1-butanol/methanol (1:1 v/v).

The recovery of ISTDs from the 1-butanol/methanol (1:1 v/v) were greater than 95% for all ISTDs except LPE (14:0) and CE (18:0) (*d*_6_) (90%), and was either similar to, or better than, the recoveries from the chloroform/methanol (2:1 v/v) or 1-butanol/methanol (3:1 v/v) methods (Figure 1). The chloroform/methanol method typically gave higher recovery than the 1-butanol/methanol (3:1 v/v) method except for Cholesterol (*d*_7_) (80% and 93%, respectively) and LPE (14:0) (80% and 87%, respectively). 

**Figure 2:**
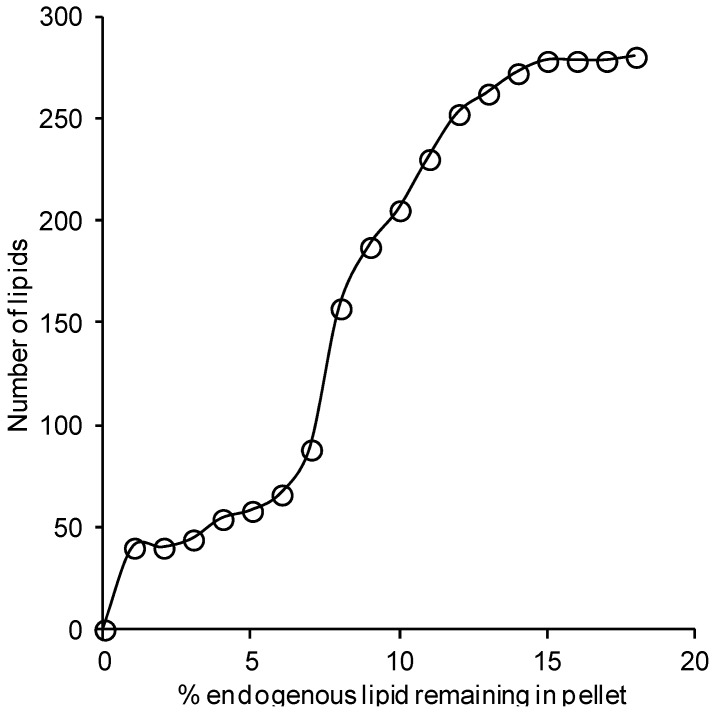
Percentage of endogenous lipids not extracted. Pellets remaining after the initial extraction (*n* = 3) were re-extracted and the lipids quantified. The re-extracted lipids were expressed as a percentage of the total lipids extracted in the first and second extractions combined.

Analysis of the re-extracted pellet showed that the amounts of re-extracted endogenous lipids were less than 18% of the sum of the initial and second extractions, and the average amount of lipid in the second extraction represented only 7.1% (median = 7.6%) of the combined total lipid (Figure 2). Out of 293 lipid species 209 (71.3%) of lipid species showed a second extraction recovery less than 10% while the highest second extraction recovery observed was related to a low abundant and neutral non-polar lipids ≈ 17%) (Supplementary Table 1).

### 2.2. Effect of Solvents on Reverse-Phase Chromatography

We have compared the chromatography when injecting a plasma lipid extracted by either 1-butanol/methanol (3:1 v/v) or 1-butanol/methanol (1:1 v/v). While the chromatographic features (retention time and peak shape) were largely the same for both extraction methods, for the most polar lipids, such as LPC 13:0, we observed both a peak broadening and an earlier elution time when the 1-butanol/methanol (3:1 v/v) extract was analyzed compared with 1-butanol/methanol (1:1 v/v) extract (Supplementary Figure 1).

### 2.3. Comparison of the Lipid Measurements with the 1-Butanol/Methanol (1:1 v/v) and Chloroform/Methanol Methods

**Figure 3:**
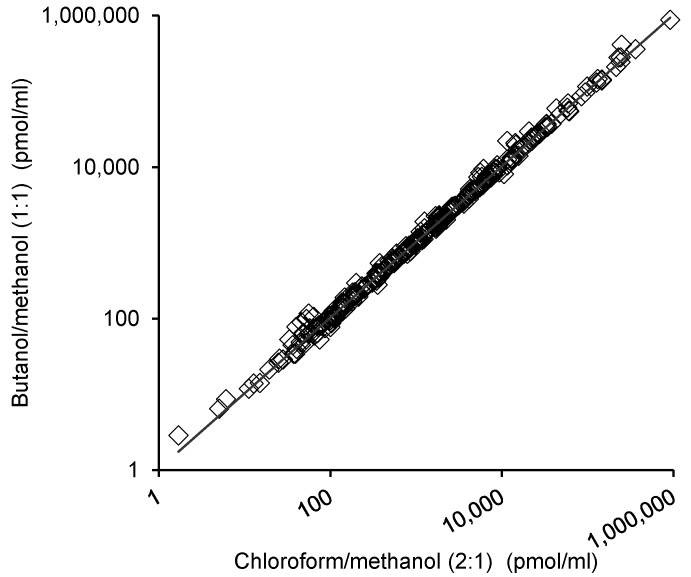
Correlation of plasma lipid measurements following different extraction procedures. Plasma (10 µL, n=10) was extracted via the 1-butanol/methanol (1:1 v/v) or chloroform/methanol methods and analyzed for 293 lipid species via liquid chromatography electrospray ionization-tandem mass spectrometry. The concentration of each lipid was calculated by comparing the area under the chromatogram with the corresponding internal standard. The concentration of each lipid determined via the 1-butanol/methanol (1:1 v/v) method was plotted against the concentration of the same lipid as determined via the chloroform/methanol method. The line of best fit was y = 1.0278x (R² = 0.976).

The comparison of the 1-butanol/methanol (1:1 v/v) extraction method with the established chloroform/methanol method was performed by comparing the lipids measurements of each method. Figure 3 shows a high correlation of the individual lipid measurements between the two methods (R^2^ = 0.976).

### 2.4. Reproducibility of the Lipid Measurements Following Extraction with Each Method 

**Figure 4:**
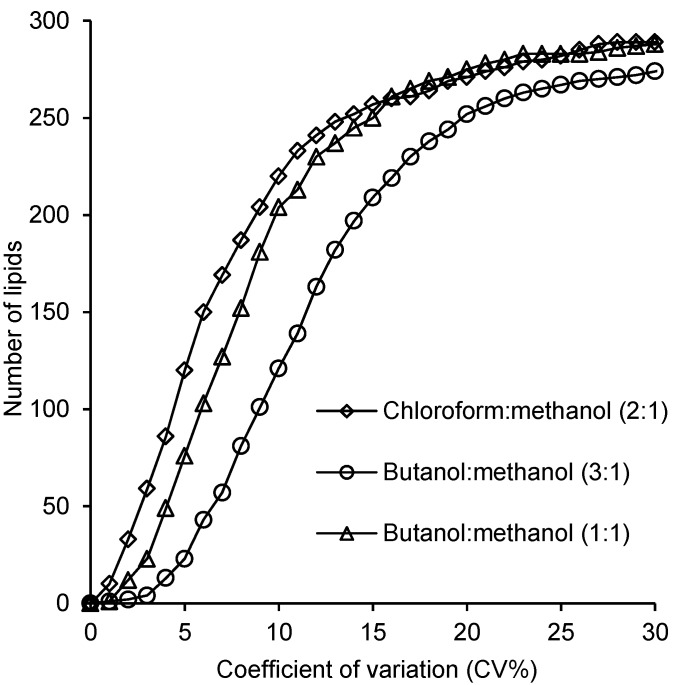
Within-batch coefficient of variation of the different extraction methods. The CV% were calculated for chloroform/methanol, 1-butanol/methanol (3:1 v/v) and 1-butanol/methanol (1:1 v/v) methods (*n* = 10). Out of 293 lipid species, 271, 252 and 275 lipid species extracted via chloroform/methanol, 1-butanol/methanol (3:1 v/v) and 1-butanol/methanol (1:1), respectively, had CV% less than 20%.

Reproducibility was evaluated by estimating intra-batch coefficient of variation (CV%) for all methods. The CV% of chloroform/methanol, 1-butanol/methanol (3:1 v/v) and 1-butanol/methanol (1:1 v/v) was calculated for (*n* = 10) pooled plasma samples of each method. These results showed that within-batch CV% were less than 20% for 271, 252 and 276 lipid species extracted by chloroform/methanol (2:1 v/v), 1-butanol/methanol (3:1 v/v) and 1-butanol/methanol (1:1 v/v), respectively (Figure 4). Highest CV% results represent mainly the variation of low abundant lipids.

**Figure 5:**
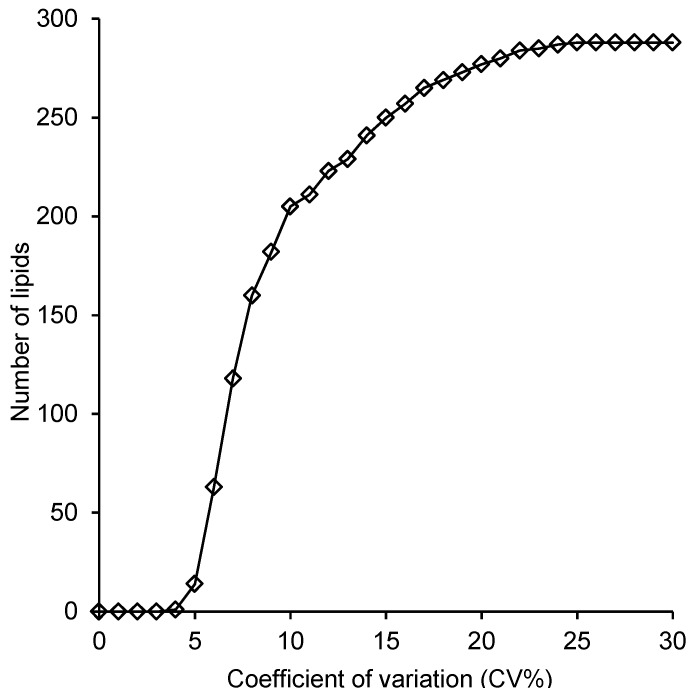
Coefficient of variation of the batch-to-batch extraction. The CV% was calculated for (*n* = 49) samples (*n* = 7/day) over two months. Out of 293 lipid species, 274 lipid species showed a CV% less than 20%.

For the 1-butanol/methanol (1:1 v/v) method, analysis of reproducibility was further extended to examine the additional variation associated with extractions performed in separate batches. Seven batches, each corresponding to seven pooled plasma samples, were extracted over a period of two months. The resulting 49 samples were then randomized and analyzed together using LC ESI-MS/MS. The resulting CV% profile (Figure 5) was broadly similar relative to the corresponding within-batch curve (Figure 4) indicating only minor increases in variation from between-batch effects. The greatest increase in variation was observed for CVs less than 5%, with little apparent difference above this. For example, there were 274 lipids with a CV < 20% in the inter-batch experiment compared with 275 for the corresponding intra-batch (Supplementary Table 1).

## 3. Discussion

Lipidomics has tremendous potential to improve our understanding of physiological, pathological and clinical conditions including type 2 diabetes, cardiovascular disease, neurological dysfunctions and cancers. Many lipid molecules have been proposed as diagnostic or prognostic markers for such conditions; however, none have thus far been translated to clinical tests. A major limitation to the discovery, validation and translation of lipid biomarkers for chronic disease is the need to conduct large population based trials. Thus, there is a need for high throughput, efficient and safe methods that can be applied in research and clinical laboratories. The 1-butanol/methanol (1:1 v/v) method presented here was evaluated for recovery, reproducibility and correlation with a recently published, but more cumbersome, chloroform/methanol method and a 1-butanol/methanol (3:1 v/v) method.

Whilst the solvent/sample ratio is limited to 20:1 for the chloroform/methanol method in order to obtain a single phase, the use of butanol/methanol is not restricted by miscibility of the solvent. Indeed, Löfgren *et al.*, found that the 1-butanol/methanol (3:1 v/v) method was linear for a wide range of sample volumes (10 to 100 μL) [23]. There was no significant difference in the recoveries of any of the lipid standards between the chloroform/methanol and the two 1-butanol/methanol extraction methods. For all internal standards the average recovery was greater than 80%. However, the recoveries of lipid standards from plasma matrix using the 1-butanol/methanol (1:1 v/v) method were either similar to, or greater than, those of the chloroform/methanol method [7] or a similar method utilizing a 1-butanol/methanol (3:1 v/v) mix. We note that the recovery of the DG (15:0/15:0) standard from the chloroform/methanol extraction appeared to be greater than 100%. This appears to be the result of acyl migration from the sn1, and sn3 positions of the 1,3-diacylglycerol to the sn2 position on the glycerol backbone during the chloroform/methanol extraction process, giving rise to 1,2- and 2,3-diacylglycerol isomers with higher response factors in the mass spectrometer. This acyl migration occurs in all extracts but appears to be greatest in the chloroform methanol extraction process, resulting in an apparent recovery of greater than 100%. For this reason, additional care should be exercised when using the chloroform/methanol extraction for diacylglycerol species. In addition to assessing recovery using spiked standards, we re-extracted the residue (pellet) remaining in the extraction microtube following the initial extraction to estimate how much of each endogenous lipid remained. Re-extraction of the pellet showed that only a small percentage of the endogenous lipid remained following the initial extraction, indicating recoveries of 82%–100% (median recovery = 92.4%). The lipid species with the poorest recoveries (82%–88%) were primarily non-polar neutral lipids (cholesteryl ester, diacylglycerol and triacylglycerol species), which are present in plasma within the hydrophobic core of lipoprotein particles. 

In comparison, the five different two phase systems recently evaluated by Reis *et al.* [24], showed significant variation in their ability to extract the less abundant lipid classes (including sphingomyelin, phosphatidylinositols, lyso-lipids, ceramides, and cholesterol sulfates) with no single method giving optimal performance for all classes. However, while single phase extraction methods offer advantages in speed, simplicity and the range of lipid classes that can be efficiently extracted, the inability to effectively remove salts and polar metabolites means that these extracts are not suitable for shotgun lipidomics but must be analyzed using LC ESI-MS/MS where the salts can be efficiently diverted to waste prior to elution of the lipids. The ability to successfully overcome the burden of salts within the LC system has been demonstrated in large cohort analyses; using 1 μL injections we are able to perform over 1000 injections before cleaning the mass spectrometer and over 4000 injections before replacement of the HPLC column (data not shown).

The ability of our method to recover endogenous lipids was either similar to, or higher than the use of single phase chloroform/methanol (2:1) [7], which was developed by our lab, the 1-butanol/methanol (3:1 v/v), which was the solvent reported recently by Lofgren *et al.* [23] and the monophasic (chloroform/methanol/water) method specifically developed for the simultaneous analysis of both polar and non-polar lipids from retina, published by Lydic *et al.* [25]. Most lipids were similar to the recovery of the single-phase chloroform/methanol (2:1) and Lofgren methods (>90% recovery), however, LPC recovery in our method showed a higher recovery than the 1-butanol/methanol (3:1) method (88% *vs.* 104%). Additionally, our method was successfully able to recover G_M3_ gangliosides (Supplementary Table 1, average = 95%), which showed lower recovery by the chloroform/methanol/water method of Lydic *et al.* (88%) [25]. For lipid extracts that are to be used for high throughput lipidomic analysis it is important that the polarity of the solvent composition allows the simultaneous solubilization of both highly polar lipids, such as lysophospholipids and gangliosides and non-polar neutral lipids such as the CEs and TAGs. Additionally, if the lipid extract is to be analyzed using reverse phase chromatography the solvent composition must be sufficiently polar so as to ensure that the most polar lipids are retained on the column, ideally having a similar polarity to the starting solvent conditions of the LC ESI-MS/MS. We observed a noticeable decrease in retention and deterioration of the peak shape of the more polar lipids when we used chloroform/methanol (2:1 v/v) and 1-butanol/methanol (3:1 v/v) (supplementary Figure 1). Indeed our previously published chloroform/methanol method required reconstitution of samples in 1-butanol/methanol (1:1 v/v) to overcome this issue [7]. The decrease in chromatography performance when samples were analyzed directly from the 1-butanol/methanol (3:1 v/v) extraction solvent is further reflected in the increased variance observed in these analyses compared to the 1-butanol/methanol (1:1 v/v) (Figure 4).

The 1-butanol/methanol (1:1 v/v) method presented here has two major advantages over our previously published chloroform/methanol based methodology. Firstly, it is significantly simpler, circumventing the need for steps involving the removal of the extraction solvent and subsequent reconstitution of the sample, significantly increasing throughput. In our hands we are able to extract more than 500 samples a day using this newer methodology. In addition, this approach avoids the use of chloroform, making it safer than methods based on halogenated solvents. Although we have not adapted this methodology to robotic extraction to date, we note that the simplicity of the methodology is likely to make it highly amenable to an automated approach.

## 4. Materials and Methods

### 4.1. Lipid Standards and Solvents

Lipid internal standards (ISTD) included species within the classes of dihydroceramide (Cer(d18:0/8:0)), ceramide (Cer(d18:1/17:0)), sphingomyelin (SM(d18:1/12:0)), lysophosphatidylcholine (LPC(13:0)), phosphatidylcholine (PC(13:0/13:0)), lysophosphatidylethanolamine (LPE(14:0)), phosphatidylethanolamine (PE(17:0/17:0)), phosphatidylglycerol (PG(17:0/17:0)), phosphatidylserine (PS(17:0/17:0)) and cholesterol (Cholesterol (*d*_7_)) were purchased from Avanti (Alabaster AL, USA). Cholesteryl ester (CE(18:0) (*d*_6_)) was purchased from CDN Isotopes (Quebec, Canada). Diacylglycerol (DG(15:0/15:0)) and triacylglycerol (TG(17:0/17:0/17:0)) were purchased from Sigma (St Louis MO, USA). Glucosylceramide (GluCer(d18:1/16:0) (*d*_3_)), lactosylceramide (LacCer(d18:1/16:0) (*d*_3_)) and trihexosylceramide (Hex3Cer(d18:1/17:0) were purchased from Matreya (Pleasant Gap, PA, USA) (Table 1). The solvents 1-butanol, methanol and chloroform were HPLC-grade and purchased from Merck KGaA (Darmstadt, Germany). Tetrahydrofuran and ammonium formate were purchased from Sigma-Aldrich (St Louis, MO, USA). 

**Table 1:** Conditions for tandem mass spectrometry quantification of major lipid species identified in human plasma.

Lipid Class	No. of species	Internal standard	Pmol ^1^	Q1 (Parent ion)	Q3 (Product Ion) ^2^	Voltage settings ^3^			
						DP	EP	CE	CXP
Dihydroceramide (dhCer)	6	Cer(d18:0/8:0)	100	[M+H]^+^	284.3	90	30	28	10
Ceramide (Cer)	6	Cer(d18:1/17:0)	100	[M+H]^+^	264.3	50	10	35	12
Monohexocylceramide (HexCer)	6	GluCer^4^(d18:1/16:0) (*d*_3_)	50	[M+H]^+^	264.3	77	10	50	12
Dihexosylceramide (Hex2Cer)	6	LacCer^4^(d18:1/16:0) (*d*_3_)	50	[M+H]^+^	264.3	100	10	65	12
Trihexosylceramide (Hex3Cer)	6	Hex3Cer(d18:1/17:0)	50	[M+H]^+^	264.3	130	10	73	12
Sphingomyelin (SM)	20	SM(d18:1/12:0)	200	[M+H]^+^	184.1	65	10	35	12
Phosphatidylcholine (PC)	46	PC(13:0/13:0)	100	[M+H]^+^	184.1	100	10	45	11
Alkylphosphatidylcholine (PC-O)	19	PC(13:0/13:0)	100	[M+H]^+^	184.1	100	10	45	11
Alkenylphosphatidylcholine (PC-P)	14	PC(13:0/13:0)	100	[M+H]^+^	184.1	100	10	45	11
Lysophosphatidylcholine (LPC)	22	LPC(13:0)	100	[M+H]^+^	184.1	90	10	38	12
Lysoalkylphosphatidylcholine (LPC-O)	10	LPC(13:0)	100	[M+H]^+^	104.1	90	10	42	5
Phosphatidylethanolamine (PE)	21	PE(17:0/17:0)	100	[M+H]^+^	NL, 141 Da	80	10	31	7
Alkylphosphatidylethanolamine (PE-O)	12	PE(17:0/17:0)	100	[M+H]^+^	NL, 141 Da	80	10	31	7
Alkenylphosphatidylethanolamine (PE-P)	11	PE(17:0/17:0)	100	[M+H]^+^	NL, 141 Da	80	10	31	7
Lysophosphatidylethanolamine (LPE)	6	LPE(14:0)	100	[M+H]^+^	NL, 141 Da	80	10	31	7
Phosphatidylinositol (PI)	16	PE(17:0/17:0)	100	[M+NH_4_]^+^	NL, 277 Da	51	10	43	14
Lysophosphatidylinositol (LPI)	4	LPE(14:0)	100	[M+ NH_4_]^+^	NL, 277 Da	80	10	31	7
Phosphatidylglycerol (PG)	3	PG(17:0/17:0)	100	[M+ NH_4_]^+^	NL, 189 Da	60	10	25	12
Cholesteryl ester (CE)	26	CE(18:0) (*d*_6_)	1000	[M+ NH_4_]^+^	369.3	30	10	20	12
Free cholesterol (COH)	1	Cholesterol (*d*_7_)	1000	[M+ NH_4_]^+^	369.3	55	10	17	12
Diacylglycerol (DG)	24	DG(15:0/15:0)	200	[M+ NH_4_]^+^	NL, NH_3_ + fatty acid	55	10	30	22
Triacylglycerol (TG)	25	TG(17:0/17:0/17:0)	100	[M+ NH_4_]^+^	NL, NH_3_ + fatty acid	95	10	30	12

^1^: Amount of internal standard per sample; ^2^: The Q3 (Product ion) corresponds to either a specific product ion or a specific neutral loss (NL); ^3^: DP = declustering potential (volts); EP = entrance potential (volts); CE = collision energy (volts); CXP = collision cell exit potential (volts); ^4^: GluCer = Glucosylcermide; LacCer = Lactosylcermaide.

### 4.2. Human Plasma

Quality control (QC) plasma was composed of pooled plasma from healthy volunteers (*n* = 6, 3 male and 3 female) aged between 20 and 45 years. This study was approved by the Alfred Human Ethics Committee and written consent was obtained from all participants. Samples were collected in ethylenediaminetetraacetic acid (EDTA) vacutainers, centrifuged at (1711 × g, 15 min, 20 °C) then pooled. To minimize the effect of oxidation, butylhydroxytoluene (BHT), 100 mM in ethanol, was added to plasma (1 µL/mL plasma), the QC aliquots were stored frozen at (−80 °C).

### 4.3. Lipid Extraction Methods

The 1-butanol/methanol (1:1 v/v) method was compared with two other single-phase methods; a one-phase chloroform/methanol method we have previously described [6], as well as a single-phase 1-butanol/methanol (3:1 v/v) modified from the method reported by Löfgren *et al.* [23]. All methods require only a single extraction.

#### 4.3.1. 1-Butanol/Methanol (1:1 v/v) Method

Plasma (10 µL) was aliquoted into a 1.5 mL eppendorf tube and 100 µL of 1-butanol/methanol (1:1, v/v) with 5 mM ammonium formate containing ISTDs (Table 1) was added. The mixture was vortexed for 10 s, sonicated for 60 min in a sonic water bath at 20 °C and then centrifuged (16,000 × g, 10 min, 20 °C). The supernatant was transferred into a 0.2 mL glass insert with Teflon insert cap for analysis by LC ESI-MS/MS.

#### 4.3.2. Chloroform/Methanol Extraction 

Plasma (10 µL) was aliquoted into a 1.5 mL eppendorf tube, then 200 µL of chloroform/methanol (2:1, v/v) was added together with 10 µL ISTD in chloroform/methanol (1:1, v/v) (Table 1). The mixture was mixed for 10 min on a rotary mixer, sonicated in a water bath (18 °C–24 °C) for 30 min, left to stand on the bench for 20 min and then centrifuged (16,000 × g, 10 min, 20 °C). The supernatant was transferred to a 96-well plate and dried under a stream of nitrogen gas at 40 °C. Samples were reconstituted with 50 µL H_2_O-saturated 1-butanol and sonicated for 10 min. Finally, 50 µL of 10 mM ammonium formate in MeOH was added. The extract was centrifuged (1700 × g, 5 min, 20 °C) and the supernatant was transferred into a 0.2 mL glass insert with Teflon insert cap for analysis by LC ESI-MS/MS.

#### 4.3.3. 1-Butanol/Methanol (3:1, v/v) Extraction

Plasma (10 µL) was aliquoted into a 1.5 mL eppendorf tube and 300 µL of 1-butanol/methanol (3:1, v/v) was added together with 10 µL ISTD in butanol/methanol (1:1, v/v) (Table 1). The mixture was vortexed for 10 s, sonicated for 60 minutes in a sonic water bath at 20 °C and then centrifuged (16,000 × g, 10 min, 20 °C). The supernatant was transferred into a 0.2 mL glass insert with Teflon insert cap for analysis by LC ESI-MS/MS.

### 4.4. Lipid Analysis

Lipid analysis was performed by LC ESI-MS/MS using an applied biosystems 4000 QTRAP triple-stage quadrupole mass spectrometer, as described previously [7]. A Zorbax C18, 1.8 µm, 50 × 2.1 mm column was used for liquid chromatography at a flow rate of 300 µL/min, using the following gradient condition: 0% solvent B to 100% over 8 min, 2.5 min at 100% B, a return to 0% B over 0.5 min and finally 0% B for 3 min prior to the next injection. Solvents A and B consisted of tetrahydrofuran:methanol:water in ratios of (20:20:60 v/v) and (75:20:5 v/v), respectively, both containing 10 mM ammonium formate. The first 1.5 min of eluent, containing the eluted salts, was diverted to waste. Multiple-reaction monitoring (MRM) in positive ion mode was performed to analyse the individual lipid species (Table 1) [7].

### 4.5. Extraction Performance

#### 4.5.1. Recovery 

The recovery of lipids for each method was determined by performing extractions where the lipid ISTDs were added prior to extraction or after extraction (and immediately before reconstitution for injection), and then comparing the relative signal intensity (area under the chromatographic peak) for each ISTD. Five replicates were performed for each analysis and samples spiked with standards pre- and post-extraction were randomized prior to analysis. The percent recovery and variance was calculated by calculating the average signal intensity in samples spiked prior to extraction as a percentage of the average signal intensity in samples spiked after extraction. To evaluate the recovery of endogenous plasma lipid species, pellets remaining after plasma extraction (*n* = 3 replicates) were re-extracted and the additional lipid quantified. The lipid in the second extraction was expressed as a percentage of the total lipids extracted in the first and second extractions combined. 

#### 4.5.2. Reproducibility 

The relative precisions of the extraction methods (combined with the mass spectrometry analysis) were assessed by determining within-batch and between-batch variation. Plasma samples (*n* = 10) were extracted and analysed to investigate within-batch variation; to investigate the between-batch extraction variation, plasma samples (*n* = 49) were extracted on seven different days (*n* = 7/day) over two months. The samples were analysed in a single LC ESI-MS/MS analysis, to reduce the effect of analytical variation (in the LC ESI-MS/MS system) that may appear between batches. Then CV% was calculated for each lipid across the seven separate extraction days.

### 4.6. Statistical Analysis

Coefficient of variation was used to evaluate the reproducibility of each method. Pearson’s correlation coefficient was used to compare the lipid measurements using the 1-butanol/methanol (1:1 v/v) method with the measurements from the chloroform/methanol (2:1 v/v) method.

## Supplementary Files

Supplementary File 1
