# Supplementary material for: An Efficient Single Phase Method for the Extraction of Plasma Lipids
Source: Metabolites. 2015 Jun 17;5(2):389–403. doi: 10.3390/metabo5020389 (PMC4495379; doi:10.3390/metabo5020389)
Supplement: Supplementary File 1 [file metabolites-05-00389-s001.pdf]

Metabolites **2015**, *5*, 389-403; doi:10.3390/metabo5020389

OPEN ACCESS

metabolites

ISSN 2218-1989

www.mdpi.com/journal/metabolites/

Article

An Efficient Single Phase Method for the Extraction of Plasma Lipids

Zahir H. Alshehry [1,2,3], Christopher K. Barlow [1], Jacquelyn M. Weir [1], Youping Zhou [1], Malcolm J. McConville [3] and Peter J. Meikle [1,2,*]

Supplementary Materials

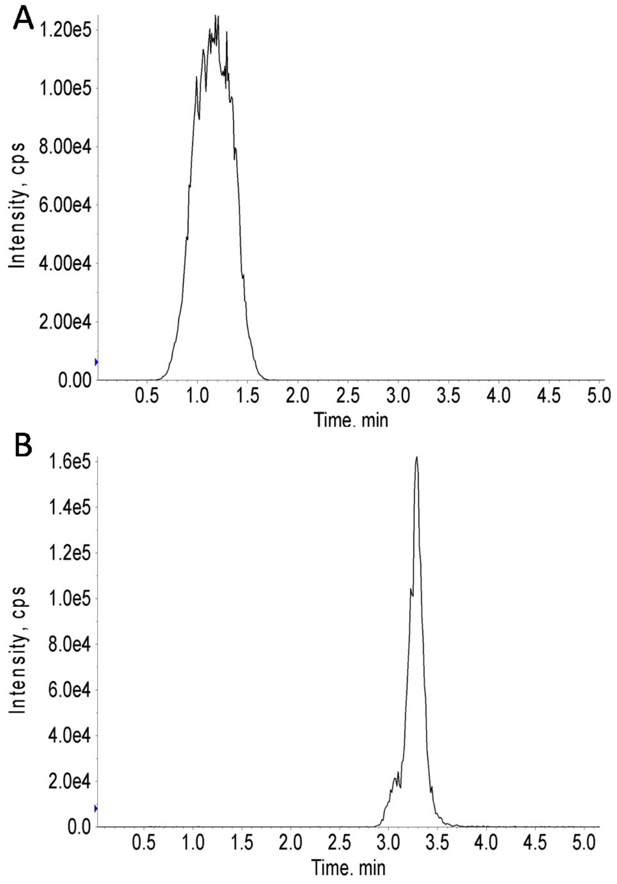

Figure S1. LPC(13:0) chromatogram for (**A**) butanol/methanol (3:1 v:v) and (**B**) butanol/methanol (1:1 v:v) methods. The early eluted internal standard LPC(13:0) shows broadening of the peak when butanol/methanol (3:1 v:v) sample was injected to the LC-MS/MS compared to butanol/methanol (1:1 v:v) sample.

Table S1. Performance of butanol/methanol (1:1) shows correlation of concentration compared to chloroform/methanol, within-batch, batch-to-batch variation and ratio of endogenous lipids after pellet's re-extraction.

Lipid	Chloroform/methanol		1-Butanol/Methanol (1:1, v:v)			
	Average pmol/mL	Within-batch CV%	Average pmol/mL	Within-batch CV%	Batch-to-batch CV%	Un-extracted endogenous lipid (%) [1]
Cer(d18:0/16:0)	40	7	40	14	16.5	0.0
Cer(d18:0/18:0)	49	13	45	20	16.6	0.0
Cer(d18:0/20:0)	13	88	24	17	16.3	0.0
Cer(d18:0/22:0)	103	9	116	13	9.9	0.0
Cer(d18:0/24:1)	102	7	113	3	10.6	0.0
Cer(d18:0/24:0)	197	10	193	2	8.6	0.0
Cer(d18:1/16:0)	299	4	306	8	8.1	8.6
Cer(d18:1/18:0)	109	3	99	3	9.9	17.8
Cer(d18:1/20:0)	97	5	94	8	7.9	2.7
Cer(d18:1/22:0)	726	4	720	7	6.3	7.9
Cer(d18:1/24:1)	948	7	973	4	6	7.6
Cer(d18:1/24:0)	2568	5	2460	4	6.3	7.8
HexCer(d18:1/16:0)	1060	7	1011	5	6.4	8.0
HexCer(d18:1/18:0)	164	15	136	9	12.3	10.1
HexCer(d18:1/20:0)	221	7	182	11	11	0.0
HexCer(d18:1/22:0)	2236	18	1870	7	7.5	6.9
HexCer(d18:1/24:1)	2180	18	1800	4	7.2	7.1
HexCer(d18:1/24:0)	3010	16	2598	4	6.5	7.2
Hex2Cer(d18:1/16:0)	4302	6	3951	4	5	7.2
Hex2Cer(d18:1/18:0)	74	11	63	19	15	0.0
Hex2Cer(d18:1/20:0)	48	11	51	13	16.8	0.0
Hex2Cer(d18:1/22:0)	264	6	257	5	8.6	3.2
Hex2Cer(d18:1/24:1)	1108	5	1046	4	7.8	7.4
Hex2Cer(d18:1/24:0)	279	9	274	7	7.3	3.5

Table S1. *Cont.*

Lipid	Chloroform/methanol		1-Butanol/Methanol (1:1, v:v)			
	Average pmol/mL	Within-batch CV%	Average pmol/mL	Within-batch CV%	Batch-to-batch CV%	Un-extracted endogenous lipid (%) [1]
Hex3Cer(d18:1/16:0)	908	10	756	10	8.9	5.9
Hex3Cer(d18:1/18:0)	94	27	98	7	16.9	0.0
Hex3Cer(d18:1/20:0)	45	8	36	17	21.8	0.0
Hex3Cer(d18:1/22:0)	170	19	180	18	13.7	3.4
Hex3Cer(d18:1/24:1)	399	7	359	5	9.5	0.0
Hex3Cer(d18:1/24:0)	193	5	182	13	11	0.0
NeuAcα2-3Galβ1-4Glcβ-Cer(d18:1/16:0)	665	3	629	3	5.8	7.2
NeuAcα2-3Galβ1-4Glcβ-Cer(d18:1/18:0)	500	10	472	12	6.1	3.2
NeuAcα2-3Galβ1-4Glcβ-Cer(d18:1/20:0)	209	9	189	15	10	0.0
NeuAcα2-3Galβ1-4Glcβ-Cer(d18:1/22:0)	512	5	494	11	7.8	2.5
NeuAcα2-3Galβ1-4Glcβ-Cer(d18:1/24:1)	744	9	712	5	8.9	8.8
NeuAcα2-3Galβ1-4Glcβ-Cer(d18:1/24:0)	548	5	512	3	12	8.5
LPC(O-16:0)	326	4	321	8	6.7	7.9
LPC(O-18:1)	206	4	200	5	8.3	6.1
LPC(O-18:0)	60	25	65	4	6.5	4.9
LPC(O-20:1)	12	9	11	8	9.7	0.0
LPC(O-20:0)	14	10	15	8	5.1	0.0
LPC(O-22:1)	26	11	25	7	7.2	0.0
LPC(O-22:0)	34	9	37	7	8.3	0.0
LPC(O-24:2)	14	22	13	31	9.4	0.0
LPC(O-24:1)	81	8	78	8	5.8	2.4
LPC(O-24:0)	63	20	56	9	6.5	6.5
LPC(14:0)	1873	3	1779	4	4	7.6
LPC(15:0)	1046	3	1039	9	4.4	7.7
LPC(16:1)	3193	2	3066	4	5.1	7.3

Table S1. *Cont.*

Lipid	Chloroform/methanol		1-Butanol/Methanol (1:1, v:v)			
	Average pmol/mL	Within-batch CV%	Average pmol/mL	Within-batch CV%	Batch-to-batch CV%	Un-extracted endogenous lipid (%) [1]
LPC(16:0)	86,558	2	84,230	6	4.6	7.7
LPC(17:1)	547	2	516	5	5.1	7.5
LPC(17:0)	1896	3	1869	6	4.6	7.3
LPC(18:3)	794	2	785	3	7.9	7.2
LPC(18:2)	33,877	2	33,876	5	5.9	7.6
LPC(18:1)	22,211	2	22,212	5	5.9	7.3
LPC(18:0)	23,559	2	23,244	5	4.1	7.4
LPC(20:5)	1130	3	1097	4	12.4	7.6
LPC(20:4)	7262	3	7330	7	11.7	7.3
LPC(20:3)	3128	2	3102	4	14.5	7.1
LPC(20:2)	492	8	397	7	14.5	7.3
LPC(20:1)	267	6	231	5	7.7	6.7
LPC(20:0)	140	2	142	8	6.3	0.0
LPC(22:6)	2101	3	2155	7	15.5	7.3
LPC(22:5)	826	2	756	5	17.3	6.8
LPC(22:1)	21	2	19	17	9.7	0.0
LPC(22:0)	29	12	28	5	8.9	0.0
LPC(24:0)	64	11	61	1	7.2	0.0
PC(28:0)	398	3	343	9	7.6	8.1
PC(29:0)	45	12	45	23	7.5	8.3
PC(30:0)	4739	6	4208	9	6.4	8.1
PC(31:0)	1280	3	1175	5	7.7	9.9
PC(31:1)	1503	4	1389	11	5.4	8.1
PC(32:0)	12,424	9	13,141	6	6.4	9.0
PC(32:1)	24,817	2	23,402	11	7	7.9

Table S1. *Cont.*

Lipid	Chloroform/methanol		1-Butanol/Methanol (1:1, v:v)			
	Average pmol/mL	Within-batch CV%	Average pmol/mL	Within-batch CV%	Batch-to-batch CV%	Un-extracted endogenous lipid (%) [1]
PC(32:2)	8014	6	7858	13	5.4	6.9
PC(32:3)	440	4	410	12	5	6.8
PC(33:0)	1878	1	1808	7	5.6	10.9
PC(33:1)	4849	4	4793	5	5.6	11.3
PC(33:2)	4453	4	4305	6	6.3	7.6
PC(33:3)	110	7	110	13	7.9	10.0
PC(34:0)	3054	6	2781	6	7.1	8.9
PC(34:1)	210,589	5	213,287	8	4.6	10.7
PC(34:2)	361,481	9	358,469	2	6	11.0
PC(34:3)	16,842	5	16,991	12	6.3	7.2
PC(34:4)	2086	6	1966	11	5.9	8.1
PC(34:5)	216	5	199	8	6.2	7.2
PC(35:0)	247	9	217	21	8.9	6.2
PC(35:1)	7931	5	7878	7	6	10.2
PC(35:2)	12,167	4	12,454	8	5.4	11.2
PC(35:3)	2064	2	1907	8	5.2	9.5
PC(35:4)	1254	2	1204	15	9	8.4
PC(35:5)	171	16	159	9	13.1	0.0
PC(36:0)	285	11	276	11	9	6.9
PC(36:1)	50,098	6	47,820	12	5.6	8.3
PC(36:2)	242,822	7	240,658	3	5.9	11.0
PC(36:3)	139,272	7	145,402	8	5	11.0
PC(36:5)	27,533	6	26,888	9	6.2	7.8
PC(36:6)	1058	5	984	10	6.5	7.1
PC(37:4)	5759	14	5865	16	6.8	11.3

Table S1. *Cont.*

Lipid	Chloroform/methanol		1-Butanol/Methanol (1:1, v:v)			
	Average pmol/mL	Within-batch CV%	Average pmol/mL	Within-batch CV%	Batch-to-batch CV%	Un-extracted endogenous lipid (%) [1]
PC(37:5)	1318	9	1281	9	6.9	10.7
PC(37:6)	606	2	547	8	6.7	8.3
PC(38:2)	8104	19	10,501	23	13	7.0
PC(38:3)	37,425	6	37,994	8	6.1	11.9
PC(38:4)	96,577	6	93,267	2	7.4	11.0
PC(38:5)	55,364	11	59,589	3	5.2	11.6
PC(38:7)	1984	3	1895	8	5.7	8.1
PC(39:5)	792	7	792	6	5.5	11.9
PC(39:6)	1795	14	1737	7	7.5	12.1
PC(39:7)	106	19	64	15	10.3	5.4
PC(40:4)	2641	6	2470	12	6.7	8.1
PC(40:5)	14,696	5	14,255	9	5.8	10.4
PC(40:6)	27,469	1	27,241	13	6.2	11.6
PC(40:7)	5130	2	5038	10	7	10.8
PC(40:8)	1405	6	1121	8	6.7	7.7
PC(16:0_20:4) [2]	143,596	9	144,891	7	5.9	12.3
PC(16:0_22:6) [2]	54,744	16	60,899	9	5.2	12.9
PC(18:1_18:3) [2]	21,114	8	20,716	16	9.2	6.5
PC(18:2_20:4) [2]	5442	15	5500	9	11.3	8.4
PC(O-32:0)	1920	2	1937	6	6.6	10.2
PC(O-32:1)	541	18	550	7	5.6	10.4
PC(O-32:2)	53	18	33	30	11.1	6.6
PC(O-34:1)	4288	0	4154	9	7	10.4
PC(O-34:2)	4973	9	4416	9	5.2	10.2
PC(O-34:3)	126	8	108	14	11.9	9.8

Table S1. *Cont.*

Lipid	Chloroform/methanol		1-Butanol/Methanol (1:1, v:v)			
	Average pmol/mL	Within-batch CV%	Average pmol/mL	Within-batch CV%	Batch-to-batch CV%	Un-extracted endogenous lipid (%) [1]
PC(O-34:4)	63	26	49	4	23.4	4.0
PC(O-35:4)	98	7	88	15	9.1	11.5
PC(O-36:2)	1620	14	1731	5	8.2	9.1
PC(O-36:3)	5168	5	5080	5	5.2	11.2
PC(O-36:4)	10,555	11	11,484	11	5.3	10.6
PC(O-36:5)	563	2	556	18	11.4	9.2
PC(O-38:4)	3829	22	4122	6	6.6	8.3
PC(O-38:5)	12,817	4	13,072	11	5.2	11.8
PC(O-40:5)	774	1	857	8	5.9	8.8
PC(O-40:6)	280	27	354	29	6.9	8.5
PC(O-40:7)	1740	9	1731	5	6	10.9
PC(P-30:0)	103	3	110	12	6.5	10.8
PC(P-32:0)	1639	4	1684	7	6	9.7
PC(P-32:1)	248	3	239	5	5.9	10.7
PC(P-34:1)	3518	1	3542	6	6.8	9.9
PC(P-34:2)	6266	8	6014	10	6.4	9.9
PC(P-34:3)	162	9	140	8	14.2	10.2
PC(P-36:2)	2094	5	2046	12	7	9.4
PC(P-36:4)	8465	5	8470	7	5.9	9.9
PC(P-36:5)	748	3	703	8	7.6	7.9
PC(P-38:4)	3093	11	2854	6	8.1	9.4
PC(P-38:5)	3318	10	3599	9	5.3	11.7
PC(P-38:6)	1122	12	1218	11	7.4	13.0
PC(P-40:6)	350	22	366	5	10.6	10.4
LPE(16:0)	1719	6	1572	3	7.8	8.7

Table S1. *Cont.*

Lipid	Chloroform/methanol		1-Butanol/Methanol (1:1, v:v)			
	Average pmol/mL	Within-batch CV%	Average pmol/mL	Within-batch CV%	Batch-to-batch CV%	Un-extracted endogenous lipid (%) [1]
LPE(18:2)	4450	1	4079	3	4.6	8.4
LPE(18:1)	3041	4	2712	7	5.3	7.6
LPE(18:0)	2331	5	2193	1	9.6	7.4
LPE(20:4)	2261	7	1685	3	6.3	8.3
LPE(22:6)	2323	1	1855	6	6	0.0
PE(32:1)	62	34	48	9	20.5	0.0
PE(32:0)	30	24	25	28	19	0.0
PE(34:3)	78	22	100	27	14.3	0.0
PE(34:2)	1123	6	1127	6	6.9	7.2
PE(34:1)	859	4	781	3	8.6	11.7
PE(35:2)	82	25	73	19	18.6	7.4
PE(35:1)	65	8	57	27	15.5	0.0
PE(36:5)	143	11	132	33	13.1	9.3
PE(36:4)	1543	6	1703	1	6.3	9.7
PE(36:3)	1082	4	1156	3	7.7	7.4
PE(36:2)	3514	5	3421	7	6.2	8.6
PE(36:1)	757	1	736	7	7	7.4
PE(36:0)	75	11	57	17	23.8	0.0
PE(38:6)	2022	4	2213	9	6.1	7.6
PE(38:5)	1669	5	1787	9	6.2	9.7
PE(38:4)	4156	5	4064	9	6.1	9.0
PE(38:3)	351	3	365	15	13.1	8.0
PE(40:7)	196	8	211	21	12.4	0.0
PE(40:6)	1080	4	1093	9	7.9	10.1
PE(40:5)	447	10	434	2	8.1	7.3

Table S1. *Cont.*

Lipid	Chloroform/methanol Average pmol/mL	Chloroform/methanol Within-batch CV%	1-Butanol/Methanol (1:1, v:v) Average pmol/mL	1-Butanol/Methanol (1:1, v:v) Within-batch CV%	1-Butanol/Methanol (1:1, v:v) Batch-to-batch CV%	Un-extracted endogenous lipid (%) [1]
PE(40:4)	84	15	78	15	17.9	0.0
PE(O-34:2)	87	27	102	13	16.4	0.0
PE(O-34:1)	127	12	139	8	13.8	3.9
PE(O-36:5)	53	31	75	10	15.7	0.0
PE(O-36:4)	718	8	722	8	7.8	11.2
PE(O-36:3)	136	1	151	16	15	3.3
PE(O-36:2)	138	8	138	5	11	5.9
PE(O-38:5)	841	4	809	9	7.7	11.3
PE(O-38:4)	596	7	590	1	7	6.4
PE(O-40:7)	224	4	193	12	11.6	8.0
PE(O-40:6)	134	5	129	14	21.7	0.0
PE(O-40:5)	158	19	144	11	12.1	0.0
PE(P-34:2)	96	12	99	12	13.5	0.0
PE(P-34:1)	77	5	73	7	16.2	5.9
PE(P-36:4)	402	1	388	9	9.7	6.7
PE(P-36:2)	262	5	250	12	9.4	4.4
PE(P-36:1)	64	14	70	20	14.6	0.0
PE(P-38:6)	318	7	343	8	9.4	3.2
PE(P-38:5)	1167	2	1177	8	6.8	9.7
PE(P-38:4)	758	3	718	4	6.9	8.5
PE(P-40:6)	250	10	248	12	11.3	3.9
PE(P-40:5)	366	9	380	13	8.9	8.7
PG(34:1)	21	35	7	173	13	0.0
PG(36:2)	34	3	39	44	19	0.0
PG(36:0)	40	10	59	16	17	0.0

Table S1. *Cont.*

Lipid	Chloroform/methanol		1-Butanol/Methanol (1:1, v:v)			
	**Average pmol/mL**	**Within-batch CV%**	**Average pmol/mL**	**Within-batch CV%**	**Batch-to-batch CV%**	**Un-extracted endogenous lipid (%) [1]**
LPI(18:2)	856	7	802	6	14.8	0.0
LPI(18:1)	893	5	802	7	18.1	0.0
LPI(18:0)	723	6	590	22	20	0.0
LPI(20:4)	1018	8	990	8	18.8	0.0
PI(32:1)	929	5	827	8	7.1	6.2
PI(32:0)	624	10	531	11	8.5	7.4
PI(34:1)	8714	1	8007	10	6.7	7.4
PI(34:0)	191	27	146	20	14.7	4.4
PI(36:4)	5088	6	4950	3	6.7	7.4
PI(36:3)	4215	1	4069	6	6.7	7.2
PI(36:2)	17,085	1	16,069	9	5.4	7.0
PI(36:1)	6809	1	6533	8	7.8	7.1
PI(38:6)	721	1	784	5	9.1	6.7
PI(38:5)	2776	12	2726	10	5.8	7.5
PI(38:4)	33,783	1	32,286	7	5.2	7.7
PI(38:3)	7712	6	6736	5	8.3	5.8
PI(38:2)	428	18	421	23	13.6	0.0
PI(40:6)	1830	8	1685	9	6.5	8.5
PI(40:5)	1804	3	1812	2	7.4	7.1
PI(40:4)	472	14	464	16	12.3	0.0
PS(36:2)	78	39	70	34	24.8	0.0
PS(36:1)	388	9	356	16	16.9	4.2
PS(38:5)	31	87	21	87	22.7	0.0
PS(38:4)	479	13	469	5	14.1	5.0
PS(38:3)	118	15	109	6	18.6	0.0

Table S1. *Cont.*

Lipid	Chloroform/methanol		1-Butanol/Methanol (1:1, v:v)			
	Average pmol/mL	Within-batch CV%	Average pmol/mL	Within-batch CV%	Batch-to-batch CV%	Un-extracted endogenous lipid (%) [1]
PS(40:6)	75	21	86	14	20.6	0.0
PS(40:5)	75	18	64	20	21.3	0.0
SM(31:1)	390	5	345	4	4.7	11.5
SM(32:2)	1155	3	1030	4	3.9	7.7
SM(32:1)	12,613	4	11,453	4	4.6	7.2
SM(32:0)	577	5	487	10	5.4	7.9
SM(33:1)	7670	5	6689	2	5.4	7.0
SM(34:3)	114	4	119	14	4.8	2.3
SM(34:2)	19,328	5	18,110	1	5.3	7.6
SM(34:1)	131,166	6	125,719	7	4.9	7.5
SM(34:0)	145,297	5	130,618	3	5.3	7.7
SM(35:2)	793	9	730	4	5.7	5.8
SM(35:1)	5004	1	4747	6	5.7	7.4
SM(36:3)	1090	3	1035	1	4.8	6.6
SM(36:2)	13,347	3	12,885	2	6.4	8.0
SM(36:1)	27,364	3	26,160	6	5.9	7.8
SM(37:2)	731	10	673	5	11.8	6.1
SM(38:2)	8533	5	9304	7	7.8	11.0
SM(38:1)	14,147	26	15,572	2	9.1	8.5
SM(39:1)	8558	2	7941	9	8	10.0
SM(41:2)	18,364	6	17,206	6	5.4	8.2
SM(41:1)	19,139	5	17,981	5	7.4	7.9
SM(42:1)	26,434	5	24,743	5	6.8	7.1
Cholesterol	879,579	10	914,021	6	9.2	7.0
CE(14:0)	24,709	12	22,122	9	9	13.9

Table S1. *Cont.*

Lipid	Chloroform/methanol		1-Butanol/Methanol (1:1, v:v)			
	**Average pmol/mL**	**Within-batch CV%**	**Average pmol/mL**	**Within-batch CV%**	**Batch-to-batch CV%**	**Un-extracted endogenous lipid (%)** [1]
CE(15:0)	19,724	6	14,503	17	9.5	12.7
CE(16:2)	1918	10	1251	7	7.3	7.1
CE(16:1)	116,050	5	97,875	4	6.7	13.6
CE(16:0)	414,059	8	248,279	16	7.3	14.0
CE(17:1)	7469	36	5795	21	13.6	9.3
CE(17:0)	9508	12	6078	12	9.6	10.2
CE(18:3)	20,823	5	14,301	4	7	7.3
CE(18:2)	281,108	7	241,255	5	8.5	14.3
CE(18:1)	279,576	6	227,198	6	7.9	13.6
CE(18:0)	22,201	8	11,497	4	6.9	7.8
CE(20:5)	29,584	3	20,622	3	8.9	6.3
CE(20:3)	5155	3	4240	14	8.4	17.1
CE(20:4)	59,554	3	4,3045	6	8.7	7.7
CE(20:2)	1566	5	1251	6	7.1	14.7
CE(20:1)	296	12	200	15	8.9	12.9
CE(22:6)	8480	6	5556	2	7.9	6.8
CE(22:5)	540	9	376	11	7.4	10.2
CE(22:4)	65	21	48	9	13.1	14.3
CE(22:1)	78	1	40	7	10.8	0.0
CE(22:0)	117	9	55	18	13.1	3.6
CE(24:6)	6	24	5	34	44.7	0.0
CE(24:5)	9	21	6	42	53.6	0.0
CE(24:4)	3	97	2	32	34.7	0.0
CE(24:1)	89	13	46	3	11.1	0.0
CE(24:0)	99	6	53	10	9.5	14.9

Table S1. *Cont.*

Lipid	Chloroform/methanol		1-Butanol/Methanol (1:1, v:v)			
	Average pmol/mL	Within-batch CV%	Average pmol/mL	Within-batch CV%	Batch-to-batch CV%	Un-extracted endogenous lipid (%) [1]
DG(14:0_18:1)	960	6	914	5	9.4	14.6
DG(16:0_16:0)	60,928	6	57,866	2	21.2	17.4
DG(16:0_18:2)	5362	6	5333	1	15.9	13.0
DG(16:1_18:1)	2792	7	2552	5	19.8	13.2
DG(16:0_18:1)	6475	2	6523	5	10.3	11.6
DG(18:1_18:3)	1016	4	947	8	15.1	11.9
DG(18:1_18:1)	9573	4	8414	7	14.4	12.2
DG(18:0_18:1)	2188	3	1857	6	17.5	14.9
DG(18:1_20:4)	1241	2	1204	11	19.8	10.4
TG(16:0_16:0_18:2)	7887	0	7021	6	6.1	13.4
TG(16:0_16:1_18:1)	37,227	2	33,222	3	6.6	13.1
TG(16:0_16:0_18:1)	10,623	1	8735	4	5.9	12.8
TG(16:0_18:2_18:2)	7273	5	6542	6	5.7	11.3
TG(16:1_18:1_18:2)	9296	3	8599	2	5.7	11.5
TG(16:0_18:1_18:2)	36,467	3	32,468	3	7.4	12.7
TG(16:0_18:1_18:1)	69,294	4	58,707	3	5.9	11.6
TG(16:0_18:0_18:1)	7417	6	5286	10	7.6	9.2
TG(18:1_18:1_18:2)	5075	7	4598	15	7.9	13.7
TG(18:1_18:1_18:1)	9696	7	8122	5	6	12.7

[1]: Pellets, remaining after extraction (n = 3), were re-extracted. The 2nd supernatant quantified and expressed as a percentage of the total lipids extracted in the first and second extractions combine; [2]: Most phospholipid signals in this analyses result from mixtures of structural isomers which are not resolved chromatographically. We report these as the sum of the carbons and double bonds in both fatty acyl chains. Where our chromatography is able to differentiate single molecular species we report these as such.

© 2015 by the authors; licensee MDPI, Basel, Switzerland. This article is an open access article distributed under the terms and conditions of the Creative Commons Attribution license (http://creativecommons.org/licenses/by/4.0/).
